# Linking ecological niches to bacterial community structure and assembly in polluted urban aquatic ecosystems

**DOI:** 10.3389/fmicb.2023.1288304

**Published:** 2023-12-15

**Authors:** Yuming Sun, Fei Ye, Qianhao Huang, Fengfeng Du, Tao Song, Haiyan Yuan, Xiaojing Liu, Dongrui Yao

**Affiliations:** ^1^Jiangsu Key Laboratory for the Research and Utilization of Plant Resources, Institute of Botany, Jiangsu Province and Chinese Academy of Sciences, Nanjing, China; ^2^Nanjing Institute of Environmental Sciences, Ministry of Ecology and Environment of China, Nanjing, China; ^3^Jiangsu Geological Bureau, Nanjing, China

**Keywords:** aquatic system, assembly process, bacterial community, ecological niches, water pollution

## Abstract

**Introduction:**

Bacterial communities play crucial roles in the functioning and resilience of aquatic ecosystems, and their responses to water pollution may be assessed from ecological niches. However, our understanding of such response patterns and the underlying ecological mechanisms remains limited.

**Methods:**

In this study, we comprehensively investigated the effects of water pollution on the bacterial structure and assembly within different ecological niches, including water, sediment, submerged plant leaf surfaces, and leaf surfaces, using a 16S high-throughput sequencing approach.

**Results:**

Ecological niches had a greater impact on bacterial community diversity than pollution, with a distinct enrichment of unique dominant phyla in different niches. This disparity in diversity extends to the bacterial responses to water pollution, with a general reduction in α-diversity observed in the niches, excluding leaf surfaces. Additionally, the distinct changes in bacterial composition in response to pollution should be correlated with their predicted functions, given the enrichment of functions related to biogeochemical cycling in plant surface niches. Moreover, our study revealed diverse interaction patterns among bacterial communities in different niches, characterized by relatively simply associations in sediments and intricate or interconnected networks in water and plant surfaces. Furthermore, stochastic processes dominated bacterial community assembly in the water column, whereas selective screening of roots and pollution events increased the impact of deterministic processes.

**Discussion:**

Overall, our study emphasizes the importance of ecological niches in shaping bacterial responses to water pollution. These findings improve our understanding of the complicated microbial response patterns to water pollution and have ecological implications for aquatic ecosystem health.

## 1 Introduction

Aquatic ecosystems, including rivers, lakes, and wetlands, maintaining global ecological equilibrium by supporting biodiversity, regulating water quality, and facilitating nutrient cycling (Biggs et al., 2016; Buonocore et al., 2021). In this context, urban rivers and lakes have emerged as indispensable components of aquatic ecosystems, closely intertwined with the daily life of human. They serve as an invaluable source of water to cities and significantly influence water cycle regulation (Hill et al., 2017; Dudgeon, 2019). However, rapid urbanization and industrial development in recent years have exerted substantial pressure on these ecosystems, compromising their ecological integrity and sustainability (Xu et al., 2019; Yang et al., 2020). Specifically, the inappropriate disposal of industrial effluents, domestic sewage, solid waste, etc. has resulted in the deterioration of water quality, degradation of biological habitats, declines of biodiversity, and even potential risks to humans (Calderon et al., 2019; Moi and Teixeira-de-Mello, 2022; Williams-Subiza et al., 2022).

Bacterial communities are crucial components of aquatic ecosystems, driving essential biogeochemical processes such as carbon, nitrogen, and sulfur cycling, and pollutant degradation (Cai et al., 2019; Zhang et al., 2020). Therefore, they are vital for sustaining the health and functionality of aquatic ecosystems. Notably, the structure and assembly of bacterial communities are highly sensitive to environmental disturbances in the water column such as water pollution, eutrophication and microplastic contaminations (Shade et al., 2011). These disturbances profoundly affect the composition and function of bacterial communities, thereby influencing the overall stability and functionality of aquatic ecosystems. For instance, Zhang et al. (2019) and Shao et al. (2023) indicated that water pollution reduces bacterial alpha diversity, while positive and non-significant results have also been documented in other studies (Wu et al., 2019; Zhang et al., 2021b). When assessing bacterial composition responses to severely pollution, Shao et al. (2023) demonstrated an increased abundance of Firmicutes and Bacteroidota phyla and a decreased abundance of Chloroflexi and Acidobacteriota phyla. These changes were accompanied by a weakening of functional genes related to microbial nitrogen and sulfur metabolism and strengthening functional genes related to carbon metabolism. Remarkably, the response of bacterial composition is influenced by the types of pollution; investigations conducted on mountain rivers revealed an increased abundance of Firmicutes and Verrucomicrobia in response to pollution (Lenart-Boron et al., 2022), whereas urban rivers exhibited the enrichment of Bacteroidetes, Verrucomicrobia, and Spirochaetes phyla (Lin et al., 2019). Furthermore, Zhang et al. (2021b) observed that the bacterial community composition and abundance of nitrogen-cycle-related genes were influenced by the sources of anthropogenic pollution, including industrial, agricultural, and domestic sources. Moreover, the influence of river pollution extent and time periods on bacterial community composition has been emphasized in previous studies (Roberto et al., 2018; Wu et al., 2019; Tai et al., 2020). These findings emphasize the sensitivity of bacterial assembly and functioning to water body pollutants, as well as their associations with pollution levels, types, and sources.

In addition to the aforementioned factors, ecological niches are crucial in shaping the responses of bacterial communities to environmental stimuli. For example, Abdullah Al et al. (2022) demonstrated that in urban freshwater ecosystems, community assembly disparities between water and sediments were more pronounced than the pollution gradients, and distinct bacterial response patterns and network associations were observed in these ecological niches. In addition to water and sediments, the abundant submerged plants in aquatic ecosystems, can offer a diverse ecological niche for microorganisms, exerting a significant influence on their community responses and functional dynamics (Hu et al., 2023). By attaching to the leaves and roots of submerged plants, microorganisms can exchange nutrients and signals with plants, significantly influencing the ecological processes within the soil and water columns. For instance, Wan et al. (2023) documented significant variations in the bacterial communities in plant compartments, such as the rhizosphere, rhizoplane, root endosphere, and leaf endosphere, of the submerged plant *Vallisneria natans*. Similar spatial heterogeneity has been documented in other aquatic plants such as *Cymodocea nodosa* and *Myriophyllum verticillatum* (Zhu et al., 2021; Banister et al., 2022). However, few studies have specifically investigated the influence of spatial ecological niches on microbial responses to pollution in aquatic ecosystems.

To address existing knowledge gaps, the present study aimed to spatially sample water column, sediments, root surfaces, and leaf surfaces from different regions of an artificial lake (control, moderate pollution and severe pollution regions). Water and sediment samples were collected from the environment and water samples from the leaf and root surfaces were collected from *V. natans*, a representative submerged plant. Subsequently, high-throughput sequencing was employed to analyze the variations and functional disparities among the bacterial communities within these samples. The primary objectives of this study were to (1) compare the effects of water pollution on the structure and function of bacterial communities within distinct ecological niches and (2) elucidate the underlying ecological mechanisms that drive the differential responses observed among these niches.

## 2 Materials and methods

### 2.1 Study sites and sample collection

Sampling was performed in July 2022 in an artificial lake (Dingjie Reservoir) in Jiangbei New Area, Nanjing, China. The area faces challenges from major pollution sources such as domestic sewage, factory sewage, contaminated surfaces, and rainfall runoff. Three representative sampling sites were selected: the inlet, outlet, and stagnant water zones of the artificial lakes, representing areas of severe pollution (SP), moderate pollution (MP), and a control area without pollution (CK), respectively (Supplementary Figure S1).

Four of water, sediment, and submerged plant samples (*Vallisneria natans*) were collected from each sampling site. The sediment samples were directly stored for subsequent microbial analyses, while the water samples experienced a two-stage filtration process: initially through a 0.45 μm filter to remove suspended particles and solid impurities, followed by filtration through 0.22 μm mixed cellulose ester membranes to concentrate microorganisms for subsequent DNA extraction. For the plant samples, leaf and root surface microbes were extracted by shaking in phosphate-buffered solution (0.1 M, pH = 7.4), followed by the detailed two-stage filtration procedure prior to microbial analyses. A total of 48 samples (3 regions× 4 ecological niches× 4 replicates) were collected for subsequent microbial sequencing analyses.

### 2.2 Chemical analysis of water samples

Water quality variables, including pH, total dissolved solids (TDS), dissolved oxygen (DO), salinity (SAL), and electrical conductivity (EC), were measured *in situ* at a depth of 20 cm below the water surface using a portable multiparameter meter (YSI ProPlus, Xylem Inc., Rye Brook, NY, USA). Afterward, water samples were collected at the same depth using a water sampler (CS-100 type Plexiglas, 2.5 L) and transferred into sterile plastic bottles. Then, the samples were analyzed in the laboratory for total nitrogen (TN) and total phosphorus (TP) content using alkaline potassium digestion and ultraviolet spectrophotometry (TN; HJ636-2012), and ammonium molybdate spectrophotometry (TP; GB11893-89), respectively (Wang et al., 2021).

### 2.3 DNA extraction and 16S rRNA gene sequencing

A total of 48 samples, including water, sediment, leaf surface and root surface samples, were sent to Biozeron Science and Technology Ltd. (Shanghai, China) for 16S rRNA gene amplicon sequencing. Genomic DNA from sediment samples was extracted directly utilizing the E.Z.N.A.® Soil DNA Kit (Omega Bio-tek, Norcross, GA, U.S.) according to manufacturer’s protocols. In contrast, the microorganism samples concentrated on the filter membranes were initially immersed in the DNA extraction solution to resuspend the microorganisms prior to subsequent DNA extraction (De Lorenzi et al., 2022; Tong et al., 2022). The V4-V5 region of the bacteria 16S ribosomal RNA gene were amplified by PCR using primers 515F (5′- GTGCCAGCMGCCGCGG-3′) and 907R (5’-CCGTCAATTCMTTTRAGTTT-3′). PCR was conducted in triplicate using a mixture containing FastPfu Buffer, dNTPs, primers, FastPfu Polymerase, and template DNA. The amplicons were then extracted and purified from 2% agarose gels using the AxyPrep DNA Gel Extraction Kit (Axygen Biosciences, Union City, CA, U.S.) following the manufacturer’s instructions. Finally, the purified PCR products were sequenced using an Illumina NovaSeq PE250 platform at Shanghai BIOZERON Co., Ltd.

The raw fastq sequences were demultiplexed, quality-filtered, and assembled in QIIME 2 (v2021), and only sequences >50 bp with an average quality score > 20 and without ambiguous characters or unidentified nucleotides were included in further analyses (Caporaso et al., 2010; Bolyen et al., 2019). Operational taxonomic units (OTUs) were clustered at a 97% sequence similarity threshold using UCLUST and the resulting OTUs were assigned a taxonomy using the SILVA (SSU138.1) 16S rRNA database with a confidence threshold of 80% (Amato et al., 2013). The sequence reads corresponding to each sample in this study can be accessed in the NCBI SRA under bioproject number PRJNA998174.

### 2.4 Statistical analyses

In this study, the influence of water pollution on water quality variables was assessed by employing SPSS (v16.0) software and the significant differences (*p* < 0.05) among treatments were determined using Duncan’s multiple range test. Then, the bacterial α-diversity was assessed using Richness and Shannon indices, which were calculated using the “vegan” package in R software and visualized with boxplots generated by the “ggplot2” package. The influence of pollution, ecological niches, and their interactions were analyzed using the nonparametric two-way Scheirer-Ray Hare test and the “rcompanion” package. Additionally, differences between treatments were evaluated using the non-parametric Kruskal-Wallis rank test (*α* = 0.05) and the “agricolae” package. The β-diversity analysis was performed using the “vegan” package based on the generated Bray-Curtis distance matrix, where the effects of pollution and ecological niches were assessed through Permutational multivariate analysis of variance (PERMANOVA). Then, the results were visualized through principal coordinate analysis (PCoA) using the “ggplot2” package.

Taxa clustering was performed based on the relative abundance of each taxon, and the correspondence between samples and species was analyzed using the online Biozeron Cloud Platform,^1^ which was then displayed via a Circos plot. Again, the effects of treatments and groups on the relative abundance of bacterial phyla were assessed using the nonparametric two-way Scheirer-Ray Hare test and the “rcompanion” package.

Bacterial community functions were predicted using the Functional Annotation of Prokaryotic Taxa (FAPROTAX v.1.2.4) database, which maps prokaryotic taxa to putative functions based on functional annotations of cultivated representatives (Merloti et al., 2019). Afterwards, “edgeR” package was employed to analyze statistical differences caused by water pollution across different ecological niches, with the criterion of False Discovery Rate (FDR) value <0.1 and fold change (FC) >2.

To investigate the interactions among microbial communities in various ecological niches, molecular ecological networks were constructed using a publicly available pipeline^2^ (Feng et al., 2022). Firstly, individual networks were created for each ecological niche utilizing random matrix theory (RMT). Only operational taxonomic units (OTUs) present in all samples within a specific network were retained for subsequent analysis. Next, a Pearson correlation matrix was generated by applying threshold values ranging from 0.01 to 0.99 with 0.01 intervals. The optimal threshold value, which resulted in a nearest-neighbor spacing distribution consistent with a Poisson distribution, was selected for network construction and the calculation of key network topological parameters such as nodes, edges, average degree, average path length, and clustering coefficient (Deng et al., 2012). Finally, the visualization of the networks was conducted using Gephi (v0.9.2) software to ensure clarity and comprehensibility of the network structures.

To evaluate the contribution of deterministic and stochastic processes in shaping bacterial community assembly, we calculated the beta nearest-taxon index (βNTI) following previously established methods (Stegen et al., 2012). βNTI quantifies ecological stochasticity using a statistical framework and a null model approach involving 999 randomizations. A value of |βNTI| >2 indicates a deterministic process, while a value of |βNTI| < 2 indicates a stochastic process. Then, according to Stegen et al. (2013) and Du et al. (2021), the βNTI and Raup-Crick index (RC Bray) based on the Bray–Curtis dissimilarity matrix were used to classify these processes into (i) heterogeneous selection, (ii) homogeneous selection, (iii) dispersion limitation, (iv) homogenized dispersion, and (v) non-dominated ecological processes.

## 3 Results

### 3.1 Comparative assessment of water quality across different sampling sites

Duncan’s multiple range test was performed to assess the changes in water quality at the different sampling sites (Supplementary Table S1). Compared with the control (CK) group sampled from undisturbed water areas, severe pollution (SP) caused a significant decrease (*p* < 0.05) in dissolved oxygen (DO) (−63.53%) and significant increase in electrical conductivity (EC) (+8.82%), total dissolved solids (TDS) (+9.21%), and salinity (SAL) (+7.14%) values. However, no significant alterations (*p* > 0.05) in these indicators were detected in samples with moderate pollution (MP). Additionally, both types of pollutions triggered a considerable decline in pH, with a more pronounced impact observed under SP conditions. When assessing the nutrient concentrations of the water samples, a progressive increase (*p* < 0.05) in total nitrogen (TN) and total phosphorus (TP) concentrations was observed with increasing levels of pollution.

### 3.2 Effects of water pollution on bacterial diversity in diverse ecological niches of aquatic environments

**Figure 1:**
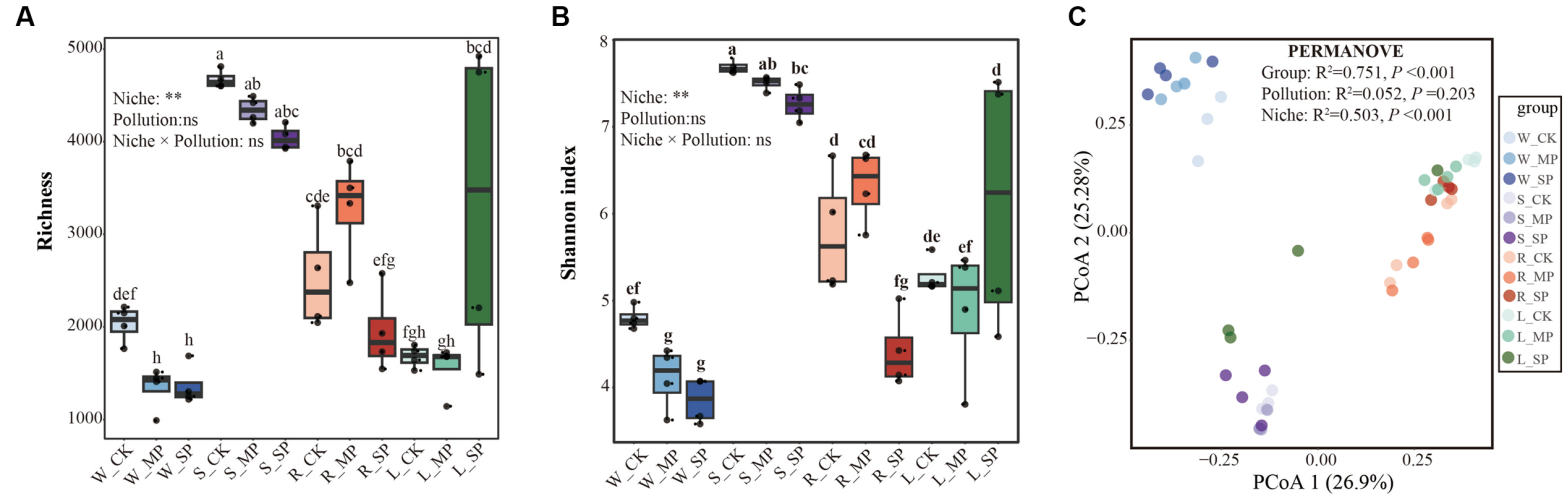
Effects of water pollution and ecological niches on the microbial diversity of bacterial communities. The boxes depict α-diversity indices, including Richness index **(A)** and Shannon index **(B)**, while the principal coordinates analysis (PCoA), **(C)** visualizes the pairwise Bray–Curtis distance between bacterial communities, with dissimilarity assessed using PERMANOVA analysis. The effects of pollution, niche and their interaction were evaluated using a non-parametric two-way Scheirer-Ray-Hare test. * and * * represent significant difference at the 0.05 and 0.01 probability levels, respectively, while ns denotes nonsignificant difference. Different letters indicate significant variations among different treatments (*p* < 0.05), as determined by the non-parametric Kruskal-Wallis rank test. W, water niche; S, sediment niche; R, root-surface niche; L, leaf-surface niche; CK, control; MP, moderate pollution; SP, severe pollution.

As shown in Figure 1, the effects of different water pollution levels on the bacterial α- and β-diversity in various ecological niches (i.e., water, sediment, root surface and leaf surface) were examined. Analysis of α-diversity variations revealed that the niches exerted a more substantial influence than water pollution. Specifically, the sediment niche exhibited higher Richness and Shannon indices than the leaf and root surface niches, whereas these values were significantly lower in the water column (Figures 1A,B). Furthermore, discrete effects of water pollution on bacterial α-diversity were observed when evaluating individual niches. In the water niche, both moderate and severe pollution led to a significant decrease in bacterial Richness and Shannon indices, whereas these indicators gradually declined with increasing pollution levels in the sediment niche. Notably, severe pollution caused the Shannon index to significantly reduce in the root-surface and increase in the leaf surface bacterial communities, compared with the control condition.

Principal coordinate analysis (PCoA) based on the Bray-Curtis distance was conducted to examine the influence of water pollution and ecological niches on bacterial community structure (Figure 1C). Permutational multivariate analysis of variance (PERMANOVA) showed a significant overall difference among the groups, which was primarily driven by the ecological niche rather than pollution treatments. Specifically, clusters associated with the water and sediment niches were fully separated from the others, whereas samples from the root and leaf surfaces showed some overlap. Furthermore, when the effects of water pollution within distinct ecological niches were considered, distinct clusters corresponding to different pollution treatments were observed in the sediment and root surface niches (Supplementary Figure S2).

### 3.3 Effects of water pollution on the bacterial community composition in different ecological niches

**Figure 2:**
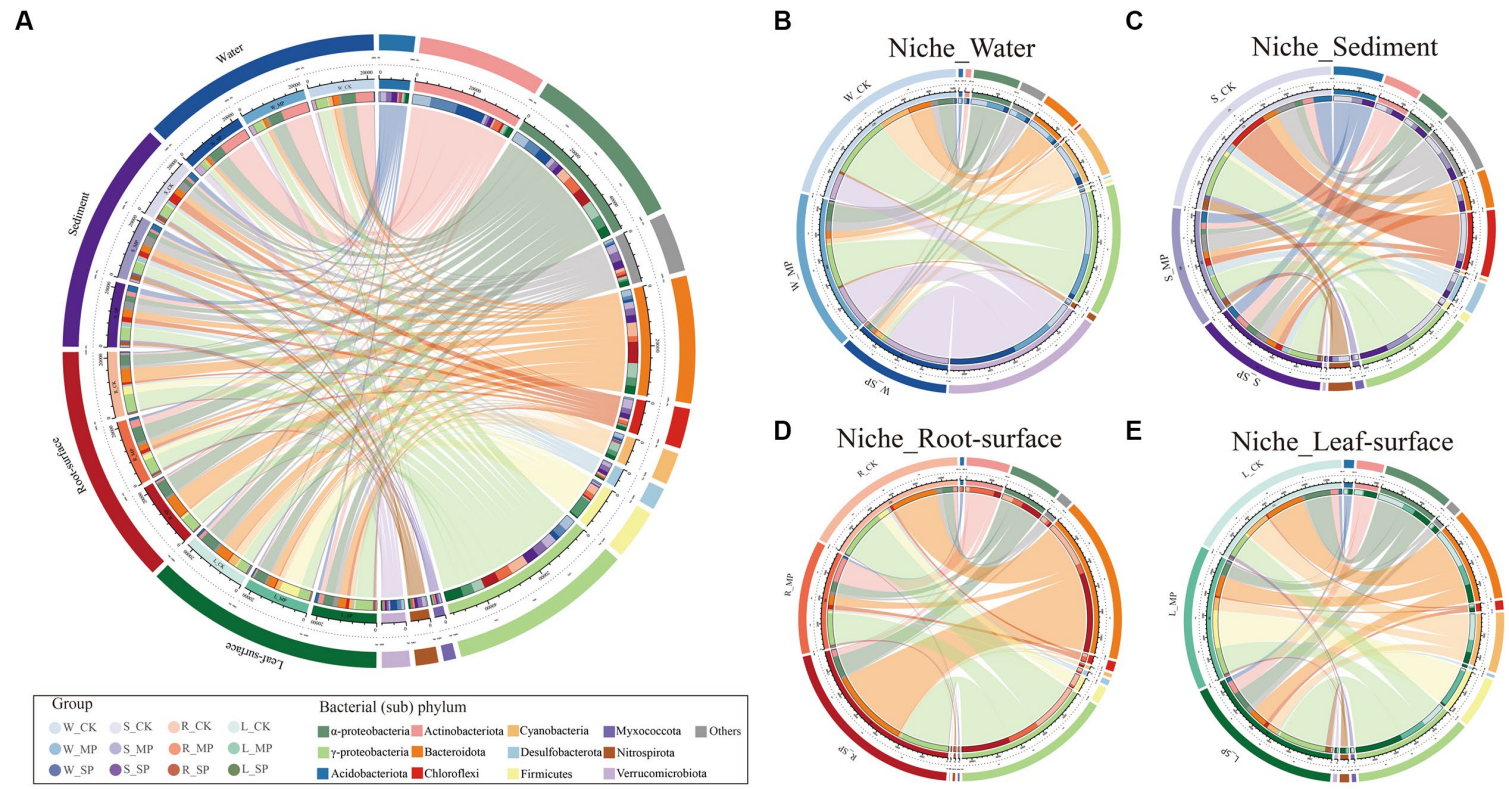
Effect of water pollution and ecological niches on the bacterial composition. **(A)** Circular visualization represents the proportional structure of the core bacterial communities at the (sub)phylum level across all samples under various pollution treatments and ecological niches. The circos plots further illustrate the taxonomic relative abundance of the responsive bacterial microbiome to pollution within specific ecological niches, including the water niche **(B)**, sediment niche **(C)**, root-surface niche **(D)**, and leaf-surface niche **(E)**. The responsive bacterial group includes bacterial OTUs that exhibit a significant response to either moderate or severe pollution, as determined by the edgeR test (FDR value <0.1 and FC > 2). W, water niche; S, sediment niche; R, root-surface niche; L, leaf-surface niche; CK, control; MP, moderate pollution; SP, severe pollution.

The community composition of various niches was analyzed to examine their response to water pollution, and variations in the dominant phyla were observed among the distinct niches (Figure 2; Supplementary Figure S3). Specifically, the relative abundance of the α-Proteobacteria phylum was significantly lower in sediments compared to other niches. Conversely, the γ-Proteobacteria phylum exhibited a consistent distribution across various niches. Additionally, the Actinobacteriota phylum was significantly enriched in water niches, whereas the Myxococcota phylum exhibited the opposite pattern. Furthermore, the Bacteroidota phylum (which was enriched in roots) and the Firmicutes phylum (which was enriched on leaf or root surfaces) were less abundant in water niches. Interestingly, Chloroflexi, Nitrospirota, Desulfobacterota, and Acidobacteriota were primarily found in the sediment, whereas the phylum Cyanobacteria mainly enriched in leaf surfaces and water niches.

When the effects of pollution on specific ecological niches were investigated, the relative abundance of Actinobacteriota increased considerably following pollution (Supplementary Figure S3). In contrast, the abundance of γ-Proteobacteria, Bacteroidota, Desulfobacterota, and Nitrospirota exhibited a decrease following pollution. Furthermore, the relative abundance of α-Proteobacteria and Bacteroidota in sediment niches gradually increases in response to water pollution, while Firmicutes show a contrasting pattern, decreasing in abundance. Within the root surface niches, an increase in the abundance of α-Proteobacteria and Bacteroidota were observed, coupled with a decrease in Chloroflexi and Myxococcota. Moreover, an increase in Nitrospirota abundance and a decrease in α-proteobacteria abundance were observed on leaf surfaces under polluted conditions.

Further screening of different niches was performed using edgeR approach (FDR = 0.01, FC = 2) to identify differentially abundant OTUs, and these bacterial taxa in various niches exhibited either shared responses or niche-specific responses to water pollution (Figures 2B–E). For instance, a significant enrichment of Actinobacteriota was observed in all niches in response to pollution. Similar results were observed in the abundance of α-Proteobacteria, except in the water niche. In addition, γ-Proteobacteria, Chloroflexi and Nitrospirota showed a post-pollution decline in the water and sediment niches, while an increase in their abundance was observed in the leaf- and root-surface niches. Interestingly, Verrucomicrobiota was exclusively enriched after pollution in the water rather than in other niches.

### 3.4 Effects of water pollution on the predicted functions of bacterial community in different ecological niches

**Figure 3:**
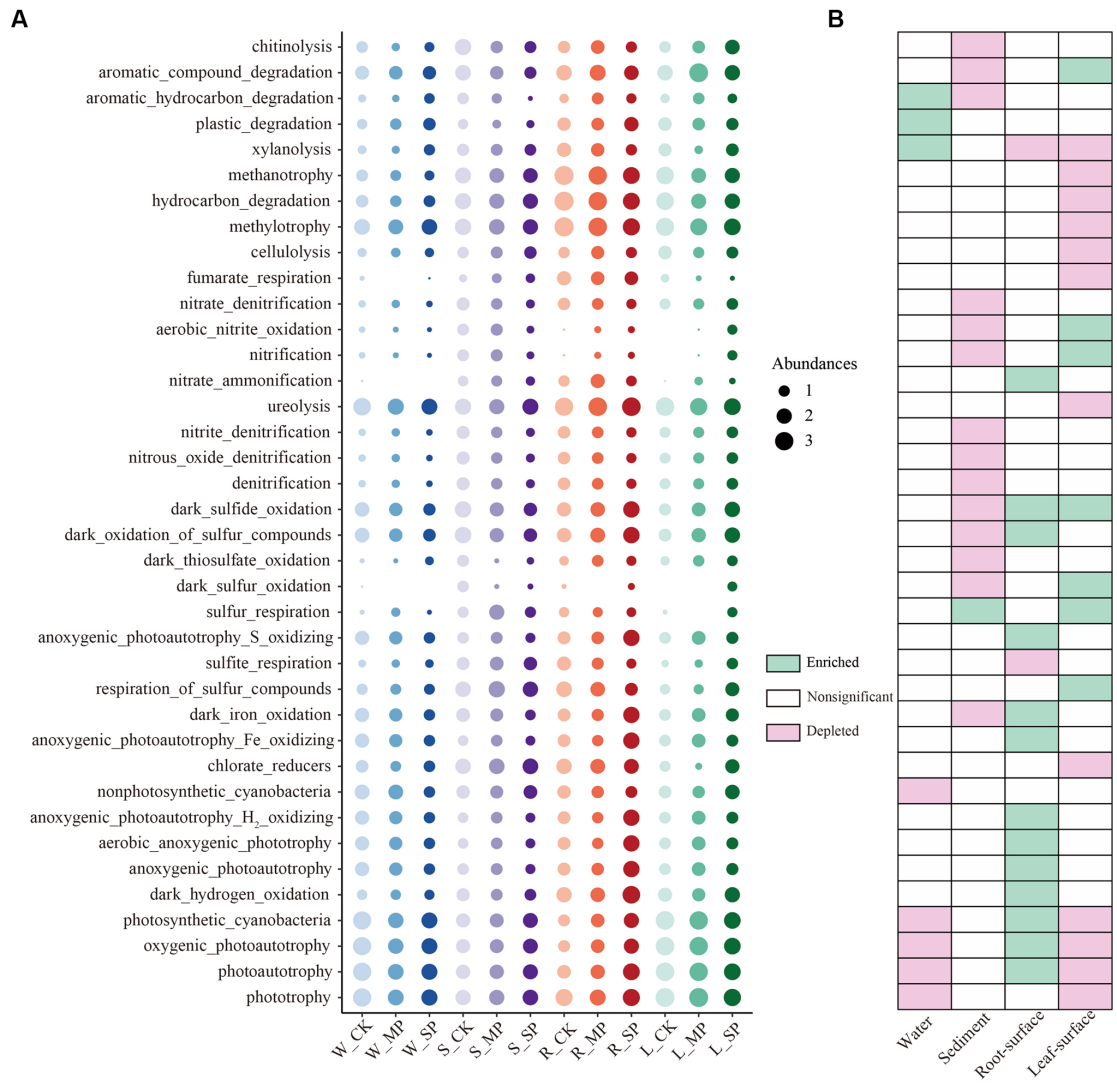
Predicted functions of bacterial community exhibiting significant responses to water pollution in distinct ecological niches. **(A)** Bubble chart illustrating the relative abundances of functional groups across all samples under different pollution treatments and ecological niches, and heatmap **(B)** displaying the variations in bacterial functions resulting from pollution across different ecological niches. Bacterial community functions showing a significant response to either moderate or severe pollution were considered significant, as determined by the edgeR test (FDR value <0.1 and FC > 2). W, water niche; S, sediment niche; R, root-surface niche; L, leaf-surface niche; CK, control; MP, moderate pollution; SP, severe pollution.

To further understand the effects of water pollution and ecological niche on bacterial function, FAPROTAX was employed for prediction and edgeR (FDR = 0.01, FC = 2) was utilized to screen the differential functions. As shown in Figure 3, functions that exhibited significant responsiveness to water pollution in at least one ecological niche were displayed. Notably, bacterial functions in both water and sediment niches were most significantly depleted owing to pollution. This depletion encompassed functions associated with nitrogen (e.g., [nitrate_denitrification], [aerobic_nitrite_oxidation] and [nitrification]) and sulfur (e.g., [dark_sulfide_oxidation], [dark_sulfur_oxidation] and [sulfur_respiration]) cycling in the sediment, as well as bacterial functions related to photosynthesis and autotrophic metabolism (e.g., [nonphotosynthetic_cyanobacteria], [photosynthetic_cyanobacteria] and photoautotrophy]) in the water niche. Functions related to organic matter degradation, such as [xylanolysis], [plastic_degradation], and [aromatic_hydrocarbon_degradation], exhibit positive responses to water pollution. On the root surface, bacterial functions associated with sulfur (e.g., [dark_sulfide_oxidation] and [dark_oxidation_of_sulfur_compounds]) and iron (e.g., [dark_iron_oxidation] and [anoxygenic_photoautotrophy_Fe_oxidizing]) cycling, as well as those linked to photosynthesis and autotrophic metabolism (e.g., [photosynthetic_cyanobacteria, [aerobic_anoxygenic_phototrophy] and [anoxygenic_photoautotrophy]) were enriched under pollution conditions. Conversely, within the leaf surface niche, functions associated with nitrogen (e.g., [aerobic_nitrite_oxidation] and [nitrification]) and sulfur (e.g., [dark_sulfur_oxidation] and [sulfur_respiration]) exhibited enrichment under pollution conditions, while those associated with carbon cycling, such as [xylanolysis], [methanotrophy], [methylotrophy], and [cellulolysis], were depleted.

### 3.5 Co-occurrence network patterns of bacterial communities in different ecological niches

**Figure 4:**
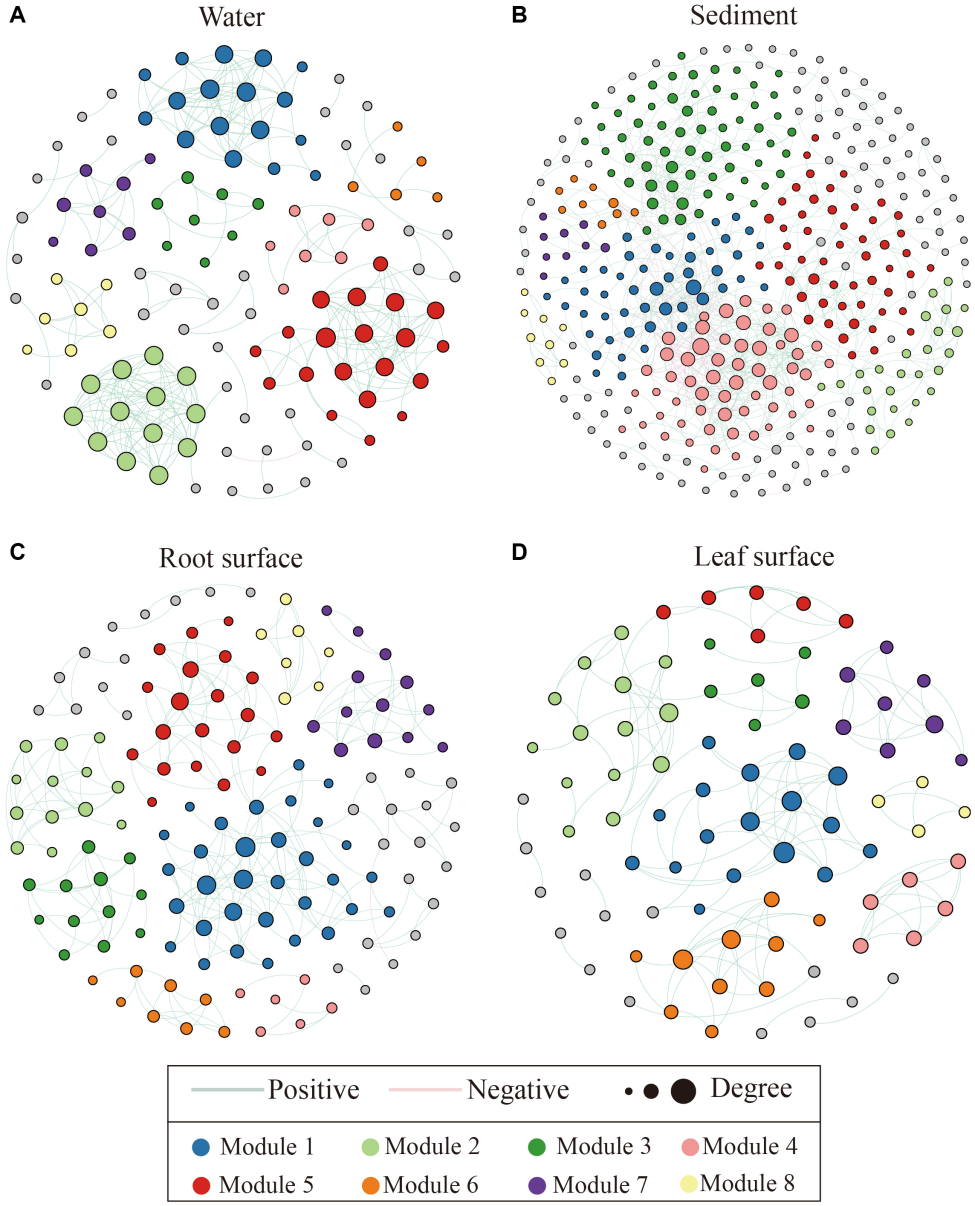
Co-occurrence network analysis of bacterial communities across distinct ecological niches, including the water niche **(A)**, sediment niche **(B)**, root surface niche **(C)** and leaf surface niche **(D)**. Nodes represent the genera present within the network, with node colors indicating their respective modules. Only the top 8 modules featuring a higher node count were colored, with the remaining modules shaded in gray. Green and red lines depict positive and negative correlations between nodes, respectively, while the size of each node corresponds to its node degree value.

Co-occurrence networks were constructed using the random matrix theory (RMT) approach for bacterial communities in different ecological niches. As depicted in Figure 4 and Supplementary Table S2, the bacterial interactions within the water niche exhibited the highest average degree and clustering coefficient, while having the lowest average path length compared with the other niches. Conversely, the sediment niche displayed the highest average path length, lowest clustering coefficient, and lowest proportion of positive interactions, indicating relative simplified associations. Furthermore, in comparison to the sediment niche, both the leaf and root surface niches demonstrated higher clustering coefficients and proportions of positive interactions, but lower average path lengths, suggesting relatively stable interactions within these bacterial communities.

### 3.6 Assembly processes of bacterial communities in different ecological niches

**Figure 5:**
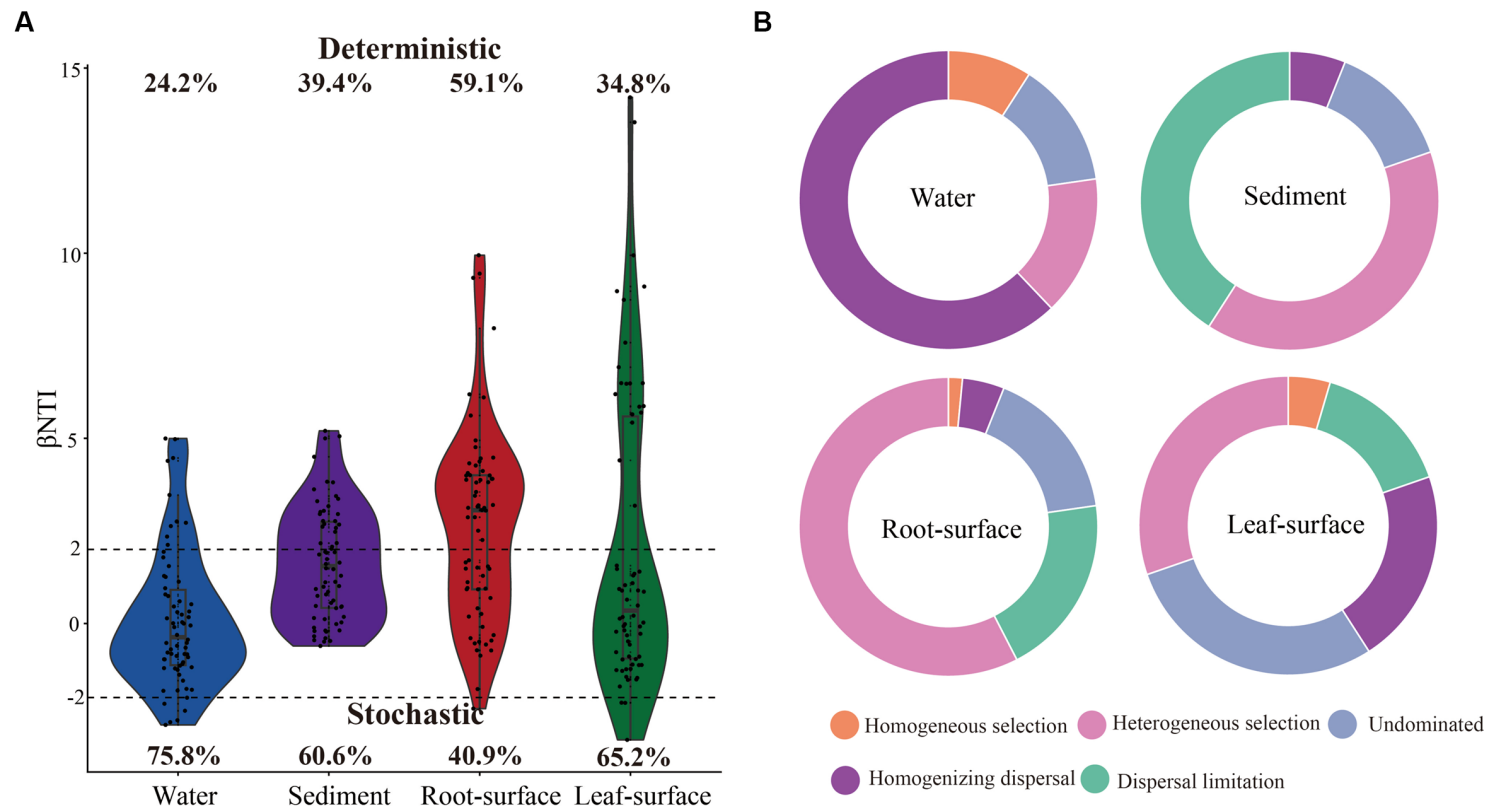
Assembly processes of bacterial communities in different ecological niches. **(A)** Violin plot showing the relative contributions of deterministic and stochastic processes in bacterial community assembly, based on the β-nearest taxon index (βNTI) values. A |βNTI| value >2 indicates a deterministic process, while a |βNTI| value <2 indicates a stochastic process. The percentages above and below the violin plots represent the proportions of deterministic and stochastic processes in bacterial community assembly, respectively. **(B)** Bar chart displaying the relative importance of five distinct ecological processes in the assembly of bacterial communities across different ecological niches.

Null-model analyses were conducted to investigate the assembly processes of bacterial communities in diverse ecological niches. As depicted in Figure 5A, stochastic processes were identified as the primary drivers of bacterial communities in the water, sediment and leaf-surface niches, whereas deterministic processes were predominant in the root-surface niche. Specifically, homogenizing dispersal, a stochastic process, exerted the greatest contribution (62.12%) to the bacterial community assembly in the water niche (Figure 5B). In contrast, in the sediment and root niches, dispersal limitation (40.91 and 19.7%, respectively), belonging to the stochastic process, and heterogeneous selection (39.39 and 57.58%, respectively), belonging to the deterministic process, played predominant roles in bacterial community assembly. Notably, in the leaf surface niche, either homogenized dispersal (21.21%) or undominated (28.79%) stochastic processes exhibited higher contributions to the bacterial communities. Furthermore, within all ecological niches, the bacterial community assembly under controlled conditions was predominantly driven by stochastic processes (Supplementary Figure S4). Nevertheless, the influence of deterministic processes on bacterial community assembly gradually increased as water pollution levels intensified (Supplementary Figure S4).

## 4 Discussion

Water pollution is expected to intensify with further urbanization and global climate change, posing detrimental consequences for the overall ecosystem stability. The diversity of bacterial communities in these aquatic ecosystems plays a critical role in maintaining ecosystem resilience (Shade et al., 2012); however, significant distinctions are proposed among bacterial communities inhabiting different ecological niches. Thus, it is important to investigate the response patterns and potential ecological mechanisms driving the behavior of bacterial communities in distinct ecological niches exposed to water pollution. Our study indicates that ecological niches affect the bacterial community structure more than pollution, as supported by the variations in bacterial community diversity and composition (Figures 1, 2; Supplementary Figure S3). Similar findings have been documented in previous studies, and the dominance of niches may be attributed to the differences in environmental characteristics and resource availability across these distinct niches (Fu et al., 2021; Zhang et al., 2021a; Abdullah Al et al., 2022). Specifically, higher bacterial Richness and Shannon indices were observed in sediments than in other niches (Figure 1), suggesting a more nutrient-rich environment and favorable microenvironmental conditions in sediments for bacterial colonization and growth (Crump et al., 2012; Li et al., 2023). In contrast, limited nutrient resources and unstable environmental conditions in the water column resulted in relatively lower bacterial diversity indices in water niches (Sheng et al., 2021; Abdullah Al et al., 2022). Despite these observations, water pollution has been observed to influence the diversity of bacterial communities within the same ecological niche, validated by the decline of α-diversity with increasing pollution levels (Figure 1). This can be attributed to bacterial inhibition by pollutants and heightened toxicity (Sun et al., 2019; Shao et al., 2023). Contrasting results were observed when evaluating microorganisms on the leaf surface (Figure 1), which suggests the role of nutrient and oxygen levels, along with the intricate interactions between leaf and microbial communities (Vandenkoornhuyse et al., 2015; Vogel et al., 2021). These findings emphasize the significance of ecological niche factors while evaluating the response of bacterial community diversity in aquatic systems, with profound implications for bacterial response to water pollution.

The bacterial community composition and function are critical factors in determining ecosystem stability. In our study, the dominant phyla in the bacterial community were Proteobacteria, Actinobacteriota, Cyanobacteria, Acidobacteriota, Bacteroidota, and Chloroflexi (Figure 2), consistent with the findings of previous studies on aquatic ecosystems (Pang et al., 2016; Boynton et al., 2017). Our research further highlights the importance of ecological niches in shaping the bacterial composition, surpassing the influence of pollution. For instance, the phyla Acidobacteriota, Chloroflexi, and Desulfobacterota exhibited higher abundance in the sediment niche compared to others, while the α-Proteobacteria phylum exhibited lower abundance (Supplementary Figure S3). The enrichment of the taxa in the sediment niche can be attributed to the availability of metabolites and nutrients (Boynton et al., 2017), whereas the limited abundance of α-Proteobacteria may be linked to their high oxygen demand, which cannot be fulfilled in the sediment environment (Shapleigh, 2011; Liu et al., 2020). Similarly, specific bacterial enrichment was observed in different ecological niches. For example, the Actinobacteriota phylum was enriched in the water niche, while Bacteroidota and Firmicutes phyla were enriched on the root and leaf surfaces (Supplementary Figure S3). These specific responses suggest that bacteria exhibit preferences for the nutritional characteristics of different ecological niches, or they may be influenced by plant-related factors. These findings indicate that bacterial community variations across distinct niches are, to a certain extent, modulated by environmental factors particularly dissolved oxygen levels and nutrient content (Qin et al., 2022; Shi et al., 2022). Abiotic factors, such as temperature and pH, are equally important in determining the bacterial assembly of different niches, which has been illustrated to have profound impacts on the survival, growth, and interactions of microorganisms (He et al., 2021; Gu et al., 2023). Consequently, further exploration of these abiotic factors is warranted in future studies to gain a deeper understanding of niche-mediated changes in the composition and functionality of microbial communities. Differences among ecological niches can also influence the response of microbial communities to water pollution. In our study, the α-Proteobacteria phylum was enriched in the root surface and sediment niches in response to pollution, partially due to the redox function possessed by certain members of the α-Proteobacteria phylum (Shapleigh, 2011). Conversely, the abundances of the γ-Proteobacteria, Chloroflexi and Nitrospirota phyla, declined in response to pollution in water and sediment niches (Figures 2B–E), indicating their sensitivity to unfavorable environments. In contrast, plant-associated niches exhibited higher abundances of the phyla, suggesting their potential role in enhancing plant resilience to pollution. Notably, the Actinobacteria phylum displayed a general sensitivity to pollution, particularly in the water niche, indicating their strong adaptability and potential for the decomposition and utilization of pollutants (Mawang et al., 2021), which is supported by their putative bacterial functions (Figure 3). In this context, the pollution-induced depletion of functions related to N and S cycling and organic matter degradation was observed in the sediment niche (Figure 3). This suggests that the biodegradation and metabolism of pollutants and nutrients are inhibited in heavily contaminated sediments, thus limiting the self-purification capacity of aquatic ecosystems (Wu et al., 2019; Li et al., 2020). Interestingly, we found positive responses in the root-surface niches for functions associated with S and Fe cycling, photosynthesis, and autotrophic metabolism (Figure 3). This indicates that the root system may promote pollutant degradation in the inter root zone and mitigate the adverse impacts on plant growth through the secretion of specific compounds that selectively promote microbial growth and functions (Singh et al., 2021; Yan H. et al., 2022). Notably, bacterial functions associated with N and S cycling were enriched on the leaf surface under pollution conditions, whereas functions related to photosynthetic C cycling, methanotrophy, and cellulolysis were suppressed (Figure 3). These observations illustrate the ecological impacts resulting from water pollution alongside plant-involved regulation of the N-S balance and shift in energy allocation and adverse impacts on specific C-cycling-related functions, due to pollutants or toxic substances (Zhang et al., 2021b; Xu C. et al., 2023). The observed ecological niche-specific responses emphasize the significance of incorporating the ecological niche context into investigations of microbial communities and their responses to environmental perturbations.

Notably, the impacts of pollution on bacterial communities were found to have a more pronounced effect in sediments rather than water environments, as indicated by the assessment of β-diversity (Supplementary Figure S2). These findings aligned with the outcomes derived from the co-occurrence networks (Figure 4), in which the bacterial communities in the water displayed more intricate and densely interconnected interactions. This observation suggests the potential facilitation of diverse ecological functions and cooperative behaviors in water niches, resulting in a higher positive correlation than that in sediment niches (Barberan et al., 2012; Abdullah Al et al., 2022). Similarly, robust positive network correlations were detected within the leaf and root surface niches (Figure 4; Supplementary Table S2). This result is consistent with previous findings and can be attributed to plant-specific recruitment or alterations in pH and hydrological features (Tylianakis et al., 2007; Shi et al., 2016). In such cases, bacterial populations may collectively respond to environmental fluctuations, engendering a positive feedback mechanism and synchronized oscillations (Deng et al., 2012; Qiu et al., 2023). Conversely, the sediment samples exhibited a higher proportion of negative correlations, indicating intense microbial competition rather than cooperation within this specific niche (Faust et al., 2012; Freilich et al., 2018). Furthermore, the co-occurrence networks of microbial communities associated with root and leaf surfaces exhibited higher clustering coefficients and lower mean path lengths than those observed in the sediment niches (Figure 4). This pattern aligns with previous studies highlighting the presence of more interconnected co-occurrence networks in aquatic plant biofilms or rhizosphere bacterial communities, which are characterized by functional cooperation facilitated by effective signal exchange and metabolic interactions (Huang et al., 2020; Liu et al., 2020). Taken together, the distinct network patterns observed in each niche underscore the ecological processes and microbial interactions that shape the specific characteristics of these communities, thereby emphasizing the significance of these niches in influencing bacterial responses.

Deterministic and stochastic processes are recognized as key factors shaping the microbial community assembly (Tilman, 2004; Zhou and Ning, 2017); however, their relative importance remains incompletely understood. In our study, we observed that stochastic processes had a more prominent influence on bacterial assembly in aquatic ecosystems, particularly within water niches (Figure 5). This observation can be attributed to chance events, such as stochastic dispersal and ecological drift, which are induced by the high mobility and environmental heterogeneity inherent in aquatic environments (Zeng et al., 2019; Tang et al., 2020; Li et al., 2023). Consequently, homogeneous dispersal, a stochastic process, made the greatest contribution to bacterial community assembly in the water niches (Figure 5B). These findings suggest that water niches are susceptible to external bacterial influx, resulting in a more homogeneous community structure. Similarly, stochastic processes, characterized by homogeneous and undominated dispersal, had a pronounced influence on the bacterial community assembly on the leaf surface (Figure 5B). These findings highlight the role of the kinetic environment on the leaf surface, which acts as a mediator for the effects of chance events and stochastic diffusion (Schlechter et al., 2019; Yan K. et al., 2022). Moreover, comparable contributions of dispersal limitations and heterogeneous selection processes to community assembly were observed in sediment niches. Dispersal limitations emphasize the spatial constraints or barriers that impede bacterial diffusion in sediments (Pan et al., 2022), whereas heterogeneous selection highlights the selective pressures imposed by diverse environmental conditions (He et al., 2022). In contrast, our findings revealed a pronounced influence of deterministic processes, specifically heterogeneous selection, on the assembly of bacterial communities on root surfaces. This indicates that plants selectively screen bacteria, influencing the stability and functioning of the root ecosystem (He et al., 2020; Shi et al., 2023). Notably, these findings suggest that stochastic processes may confer benefits to the co-occurrence networks of the bacterial community (Figures 4, 5), as reported by previous studies investigating straw incorporation (Xu Z. et al., 2023), straw decomposition (Wahdan et al., 2023), and plant invasion (Chen and Wen, 2021). Considering the influence of water pollution, the assembly of bacterial communities across different niches was primarily governed by stochastic processes under unpolluted conditions, whereas the selective pressures exerted by water pollution increased the influence of deterministic processes (Supplementary Figure S4). These results align with those of previous studies and underline the role of specific environmental conditions, selective pressures, or pollutant drivers in bacterial assembly (Tai et al., 2020; Wang et al., 2022; Zhang et al., 2022). Overall, these findings emphasize the significance of understanding the interactions between deterministic and stochastic processes in unraveling microbial community dynamics, as well as the role of ecological niches and environmental factors in driving their assembly.

## 5 Conclusion

Our study demonstrated that the influence of ecological niches on bacterial community structure in artificial lakes surpass the direct effect of pollution. This was evident in the disparities observed in bacterial diversity, composition, and function. We also observed distinct species interactions and community assembly processes in different niches, driven by stochastic or deterministic factors. This indicated their adaptations to nutritional characteristics or plant-derived compounds. Moreover, these niche-driven differences also affected bacterial responses to water pollution. The responsiveness of bacterial taxa and their predicted functions exhibited considerable variation among the different ecological niches, suggesting a combined effect of niches and environmental variability. These findings advance our understanding of bacterial responses to water pollution and their relationship with different niches. Thus, they significantly impact the prediction of bacterial community variations and effective ecosystem management.

## Data availability statement

The datasets presented in this study can be found in online repositories. The names of the repository/repositories and accession number(s) can be found at: https://www.ncbi.nlm.nih.gov/, PRJNA998174.

## Author contributions

YS: Formal analysis, Writing – original draft. FY: Conceptualization, Writing – original draft. QH: Validation, Writing – original draft. FD: Formal analysis, Writing – original draft. TS: Validation, Writing – original draft. HY: Formal analysis, Writing – original draft. XL: Conceptualization, Writing – review & editing. DY: Conceptualization, Writing – review & editing.

## Footnotes

^1^ http://www.cloud.biomicroclass.com/CloudPlatform

^2^ http://mem.rcees.ac.cn:8081
