# Supplementary material for: Linking ecological niches to bacterial community structure and assembly in polluted urban aquatic ecosystems
Source: Front Microbiol. 2023 Dec 15;14:1288304. doi: 10.3389/fmicb.2023.1288304 (PMC10754954; doi:10.3389/fmicb.2023.1288304)
Supplement: Supplementary file 1 [file Data_Sheet_1.pdf]

Title：Linking ecological niches to bacterial community structure and assembly in polluted urban aquatic ecosystems

Table S1 The water quality variables at different sampling sites

	CK	MP	SP
DO (ppm)	7.76±0.03b	7.84±0.04a	2.83±0.06c
EC (μS/cm)	349.05±0.19b	348.20±0.32b	379.85±5.16a
TDS (mg L^{-1})	192.48±0.19b	187.20±18.37b	210.20±3.42a
SAL (%)	0.14±0.00b	0.14±0.00b	0.15±0.01a
pH	9.42±0.00a	8.97±0.02b	8.00±0.01c
TN (mg L^{-1})	0.28±0.02c	0.49±0.05b	0.57±0.07a
TP (mg L^{-1})	0.06±0.00c	0.08±0.01b	0.09±0.01a

Data are presented as the means ± standard deviation (SD) of six replications. Different letters in the same line indicate a significant difference according to Duncan's multiple range test ($P < 0.05$).

CK: control; MP: moderate pollution; SP: severe pollution.

DO: dissolved oxygen; EC: electrical conductivity; TDS: total dissolved solids; SAL: salinity; TN: total nitrogen; TP: total phosphorus.

Table S2 Topological parameters of bacterial interaction networks of different ecological niches

	Water	Sediment	Root-surface	Leaf-surface
Nodes	122	381	140	85
Edges	298	883	284	159
Positive edges (%)	99.66	86.52	99.65	100
Negative edges (%)	0.34	13.48	0.35	0
Average degree	4.885	4.635	4.057	3.741
Average path length	1.863	6.325	3.502	3.123
Clustering coefficient	0.791	0.395	0.612	0.667

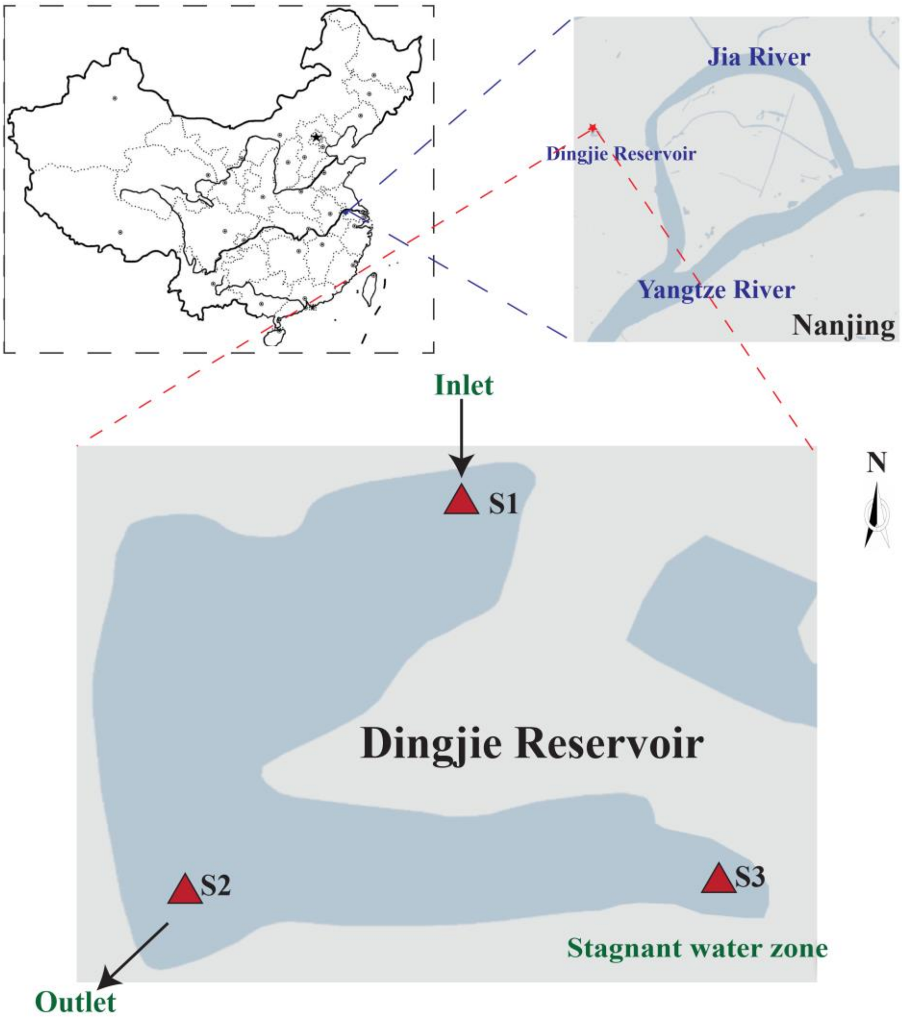

Fig. S1 Map showing the location and distribution of sampling sites throughout the Dingjie Reservoir region in Nanjing city. The sites S1, S2, and S3, distinguished by red triangles, denote the inlet, outlet, and stagnant water zone of the reservoir. These sampling sites correspond to severe pollution, moderate pollution and a control area, respectively.

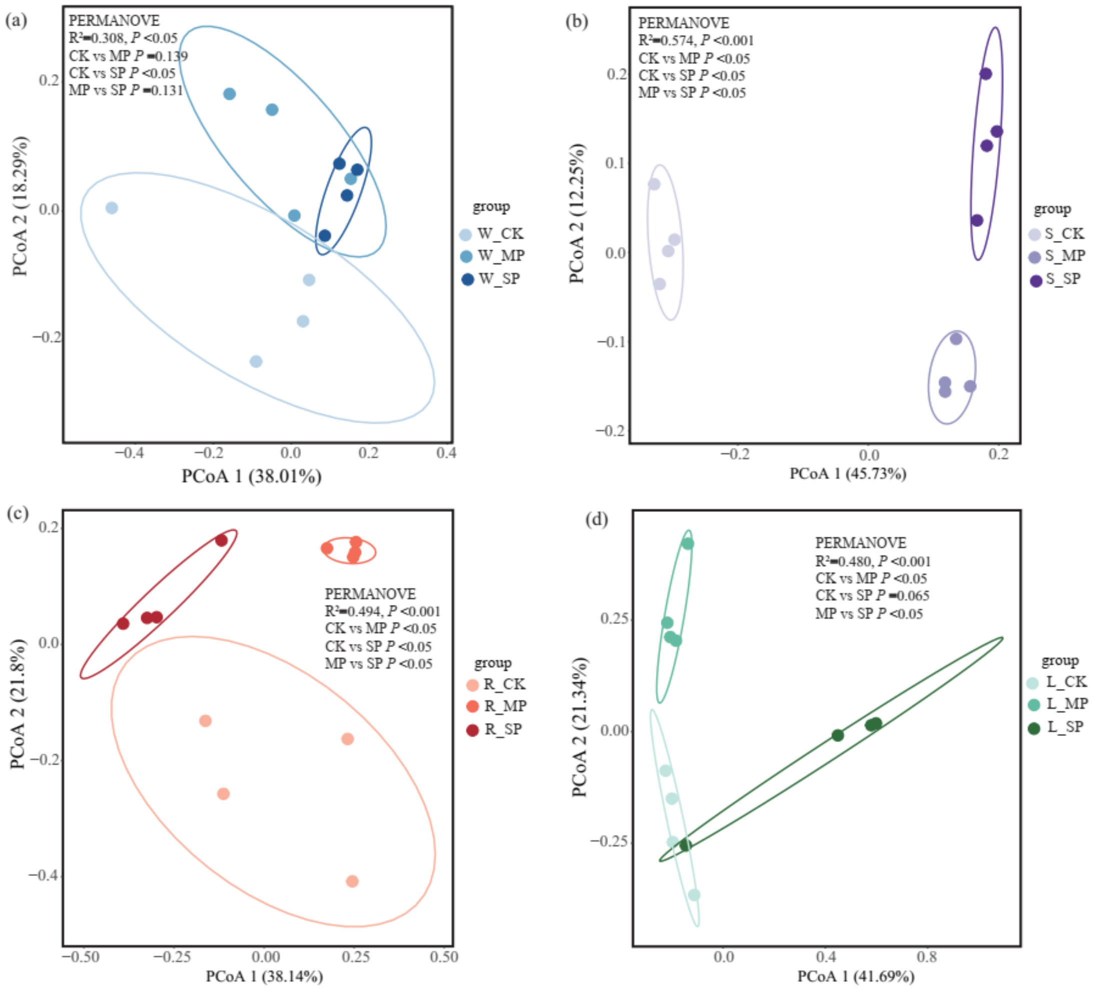

Fig. S2 Effect of water pollution on the Bray-Curtis distance between microbiome samples in individual ecological niches, including water (a), sediment (b), root-surface (c) and leaf-surface (d). The differences between different treatments were assessed using PERMANOVA analysis.

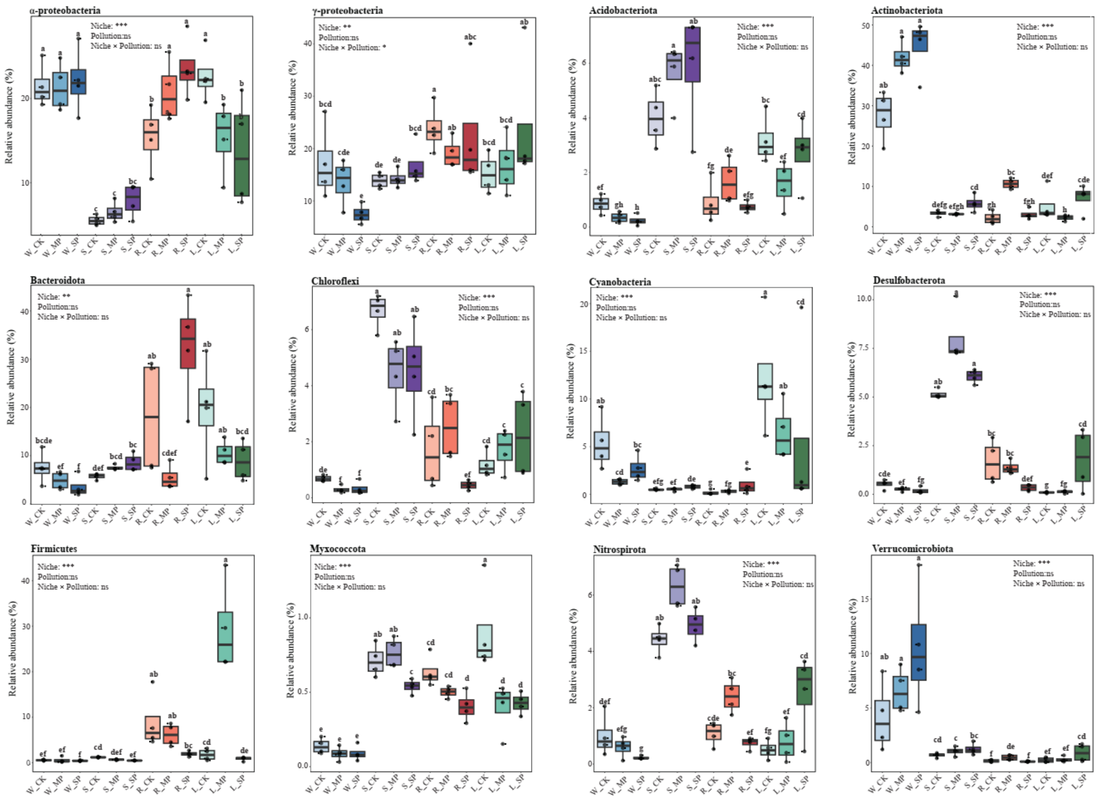

Fig. S3 Effects of water pollution and ecological niches on the relative abundance of different bacterial (sub) phyla.

The effects of pollution, niche and their interaction were evaluated using a non-parametric two-way Scheirer-Ray-Hare test. * and * * represent significant difference at the 0.05 and 0.01 probability levels, respectively, while ns denotes nonsignificant difference. Different letters indicate significant variations among different treatments (P< 0.05), as determined by the non-parametric Kruskal-Wallis rank test.

W: water niche; S: sediment niche; R: root-surface niche; L: leaf-surface niche; CK: control; MP: moderate pollution; SP: severe pollution.

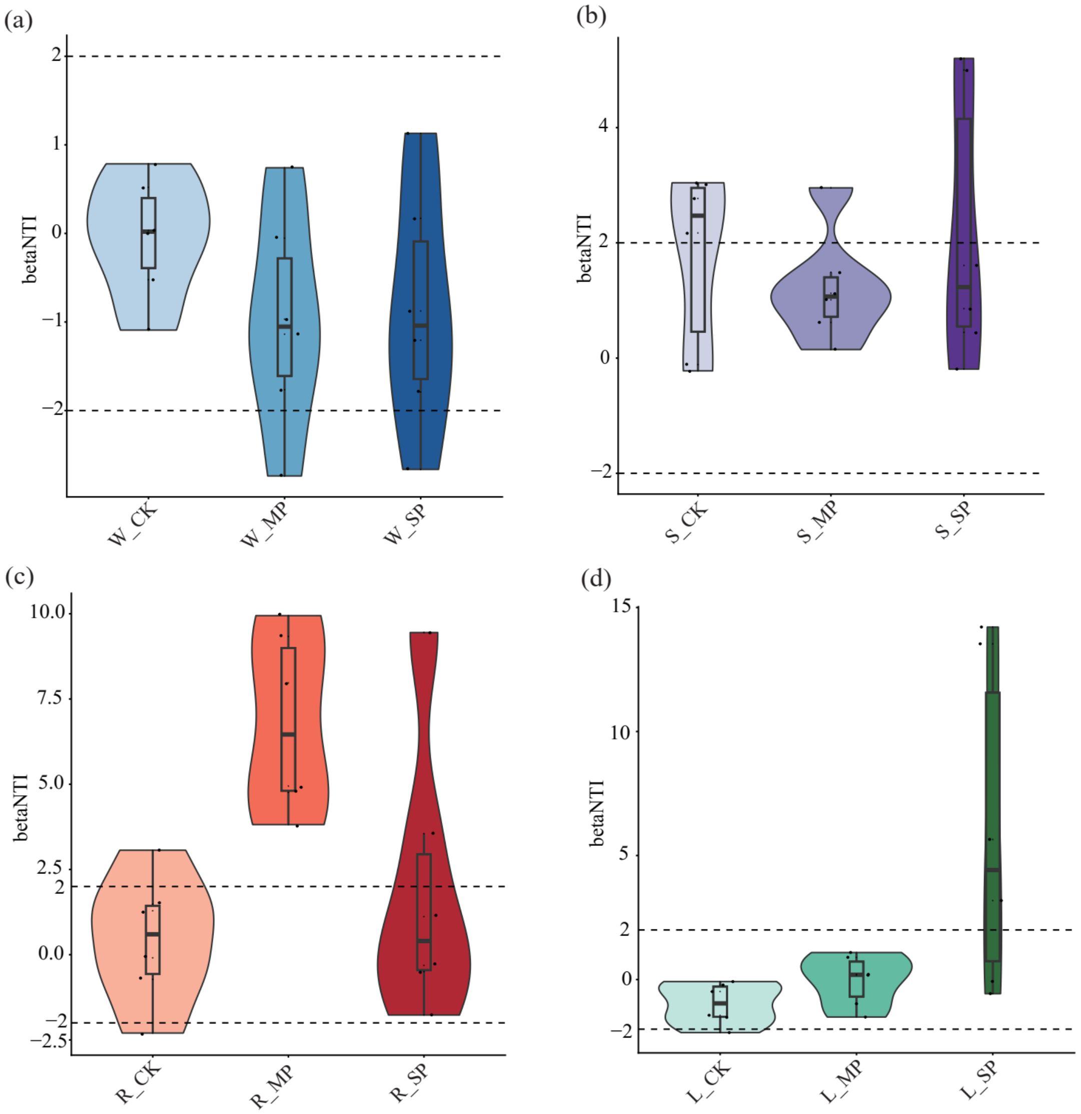

Fig. S4 Violin plot showing the influence of water pollution on the relative contributions of deterministic and stochastic processes in bacterial community assembly in different ecological niches (a, water; b, sediment; c, root-surface and d, leaf-surface), based on the β-nearest taxon index (βNTI) values. A |βNTI| value >2 indicates a deterministic process, while a |βNTI| value <2 indicates a stochastic process.
